# A randomised controlled trial comparing the effectiveness of surgical and nonsurgical treatment for cervical radiculopathy

**DOI:** 10.1186/s12891-020-3188-6

**Published:** 2020-03-16

**Authors:** Mirad Taso, Jon Håvard Sommernes, Frode Kolstad, Jarle Sundseth, Siri Bjorland, Are Hugo Pripp, John Anker Zwart, Jens Ivar Brox

**Affiliations:** 1 Department of Physical Medicine and Rehabilitation, Oslo University Hospital HF, Ullevål, Postboks 4956, Nydalen, 0424 Oslo, Norway; 2 Department of Neurosurgery, Oslo University Hospital, Rikshospitalet, Oslo, Norway; 3 Faculty of Medicine, University of Oslo, Oslo, Norway; 4 Oslo Centre of Biostatistics and Epidemiology, Research Support Services, Oslo University Hospital, Oslo, Norway; 5 Research and Communication Unit for Musculoskeletal Health, Division of Clinical Neuroscience, Oslo University Hospital, Oslo, Norway

**Keywords:** Cervical radiculopathy, Treatment, Surgery, Anterior cervical decompression and fusion, Nonsurgical, Physical medicine and rehabilitation, Effectiveness, Shared decision making, RCT

## Abstract

**Background:**

Cervical radiculopathy is usually caused by disc herniation or spondylosis. The prognosis is expected to be good in most patients, but there is limited scientific evidence on the indications for nonsurgical and surgical treatments. The aim of the present study is to evaluate and compare the effectiveness of surgical and nonsurgical treatment in two trials – including disc herniation and spondylosis, respectively, and to evaluate factors that contribute to better decision making.

**Methods/design:**

Patients with disabling radicular arm pain and MRI-proven cervical disc herniation or spondylosis will be randomised to receive nonsurgical or surgical treatment. The follow-up period is one year and the sample size is estimated to be 50 for each arm in the two trials, giving a total of 200 patients. The primary outcomes are the Neck Disability Index and arm pain. Secondary outcomes include neck pain; EQ-5D and costs to evaluate cost-effectiveness; prognostic factors; CT and MRI scans, to estimate intervertebral foraminal area and nerve root compression; and the expected minimal improvement for willingness to undergo treatment.

**Discussion:**

The outcomes of this study will contribute to better decision making in the treatment of cervical radiculopathy.

**Trial registration:**

This study has been registered at ClinicalTrials.gov as NCT03674619, on September 17, 2018.

## Background

Neck pain is among the leading causes of disability worldwide [1]. The yearly prevalence is 48, and 25% of women and 13% of men reported that they suffered from neck pain weekly in two Norwegian epidemiological surveys [2, 3]. Classifying neck pain in those who consult healthcare providers is challenging. Interpreting images is difficult because of the high frequency of degeneration in asymptomatic populations [4]. For example, in a systematic review, disc protrusion was reported in 29% of 20-year-old asymptomatic persons, and in 43% of 80-year-olds, while facet joint degeneration was reported in 4 and 83%, respectively [4].

In contrast, the yearly prevalence of cervical radiculopathy is relatively low. It was estimated to 83/100000, peaking in fourth and fifth decades, in a large epidemiological study applying wide criteria, including neck and arm pain, and corresponding MRI findings indicating that one or more nerve roots were affected [5]. Cervical radiculopathy is caused by disc herniation, spondylosis, or a combination of herniation and spondylosis. In 80% of patients, the C6 or C7 roots are affected [5]. Cervical spondylosis refers to degenerative changes that occur in the cervical spine with age, most often manifesting as decreased disc height and hypertrophy of the intervertebral joint. Symptoms usually develop more gradually than the sudden, intense arm pain reported by patients with disc herniation.

Cervical disc herniation and spondylosis may trigger local ischemia and inflammation, mediated by biochemical and immunological factors that contribute to the pathophysiology of radiculopathy [6]. High levels of IL-6 were found to be a predictor of slow recovery in patients with lumbar radicular pain [7].

The natural course of cervical radiculopathy is difficult to outline. Recent studies have described a favourable course at an average of 6 months, with complete recovery ranging from 24 to 36 months [8, 9]. There is limited evidence on prognostic factors; however, durations greater than 6 months, higher pain scores, radicular signs, psychosocial factors, sickness absences, and surgery-related factors are reported to be associated with poorer outcomes [9, 10]. Most patients with cervical radiculopathy are treated nonsurgically. The effectiveness of different nonsurgical treatments in comparison to placebo or the natural course is not known. A recent randomised controlled trial (RCT) compared cervical collar, physiotherapy, and a wait-and-see policy in patients with cervical radiculopathy lasting 3 weeks or more [11]. With any of the three interventions, mean arm pain intensity decreased from about 70% of the worst possible pain at baseline to about 20% of the worst possible pain at 6 months follow-up [11]. Improvement in condition at 6 and 12 weeks was significantly better in those who received physiotherapy or a collar. In the aforementioned epidemiological study, 561 patients were studied for 5 years, and 90% were asymptomatic or only mildly incapacitated owing to cervical radiculopathy. Twenty-six per cent of the patients underwent surgery. The strongest predictor of surgery was a combination of radicular pain, sensory loss, and muscle weakness, yielding a hazard ratio of 17.3 in a multivariable model.

The most common surgical treatment is discectomy and fusion [12]. Anterior cervical discectomy is one of the most frequently performed spinal procedures; in the United States, almost 550,000 patients were operated on between 2005 and 2008 [13]. The surgical rates for cervical radiculopathy are lower in Norway compared to the United States, but increased by 86.5% from 2008 to 2014, and was 2.5 times higher in counties with the highest rates compared to those with the lowest rates [14]. Neither the observed increase over time nor geographical differences are likely to be explained by variations in the prevalence of cervical radiculopathy. A recent review stated that previous studies have led most investigators to conclude that cervical radiculopathy is a self-limiting phenomenon in most cases [15]. This directly contradicts the increasing surgery rates.

The success rates of surgical interventions are reported to range between 80 and 95% [16]; however, two systematic reviews (SRs) have found no clear benefits of surgery over nonsurgical treatments [17, 18]. One of the SRs included two small RCTs [19–21], the other included six additional controlled clinical trials. The evidence that can be drawn from these systematic reviews is considered to be limited. Results suggest that selection criteria, observer bias, the natural course and placebo mechanisms play an important role in the reported high success rates after surgery. Further, the disc prosthesis (cervical arthroplasty) has been proposed to improve results. A recently published Norwegian multicentre trial did not favour discectomy and disc prosthesis compared with the traditional discectomy and fusion [22]. The trial included patients with one-level radiculopathy (C6 or C7) primarily caused by spondylosis. The success rate was 75%, estimated by the number of patients reaching the minimal clinical important change (MCIC). However, reported values of the minimal important clinical change of the Neck Disability Index (NDI) differ largely [23]. A systematic review reported that MCIC varied from 10 to 38, on a scale from 0 to 100 [24]. An MCIC of 10 is most commonly used in trials comparing various surgical procedures for cervical radiculopathy [22]. The global perceived change is often used as an external criterion for estimating MCIC; however, the improvement expected by the patient in order to undergo surgery or non-operative treatments has not been examined, to the best of our knowledge.

There is limited knowledge on the indications for surgery in patients with pain syndromes in general, and particularly in patients with cervical radiculopathy caused by disc herniation. It is believed that the prognosis of non-operative treatment is better in patients with disc herniation than in patients with spondylosis, but there is a lack of clinical trials to corroborate this. One reason for this is that a strict classification based on MRI-findings has been difficult to obtain. Surgery is conducted to decompress the nerve root, but the correlation between the intervertebral foraminal area, root compression, and symptoms has been poorly investigated, and findings are questionable because neither the measurement error nor the inter-rater agreement of findings has been reported. Albert et al. found no postoperative correlation between surgical graft height and symptom relief [25]. The intervertebral foraminal area of different segments of the cervical spine correlates with disc height and disc degeneration – measured using CT or MRI scans [26] – and is increased by cervical flexion [27]. More studies have been performed on the lumbar spine. A recent study found moderate inter-rater reliability for evaluation of postoperative nerve-root thickening and compression, and that postoperative compression or dislocation observed in patients operated for disc herniation was not correlated significantly to the outcome [28]. Therefore, assessing the association of the neuroforaminal area and nerve root compression in the cervical spine, including a methodological evaluation, is warranted.

Further research may improve our understanding and reverse or limit current practice. Forty-two per cent of practices believed to be effective, were reversed according to a systematic review evaluating trials published in a high impact journal over a 10-year period [29]. For example, a few years ago, spinal surgeons and radiologists strongly believed that vertebroplasty for osteoporotic vertebral fractures was very effective, but two sham-controlled trials found that it was not more effective than placebo [30, 31].

The current study aims to evaluate the effectiveness of surgical and nonsurgical treatment in two separate trials on patients with disc herniation and spondylosis, respectively. The trials will be merged to evaluate cost-effectiveness, prognostic factors, radiology, and expected outcome. The studies are likely to contribute to better evidence for the treatment of cervical radiculopathy.

### Aims

**Figure 1:**
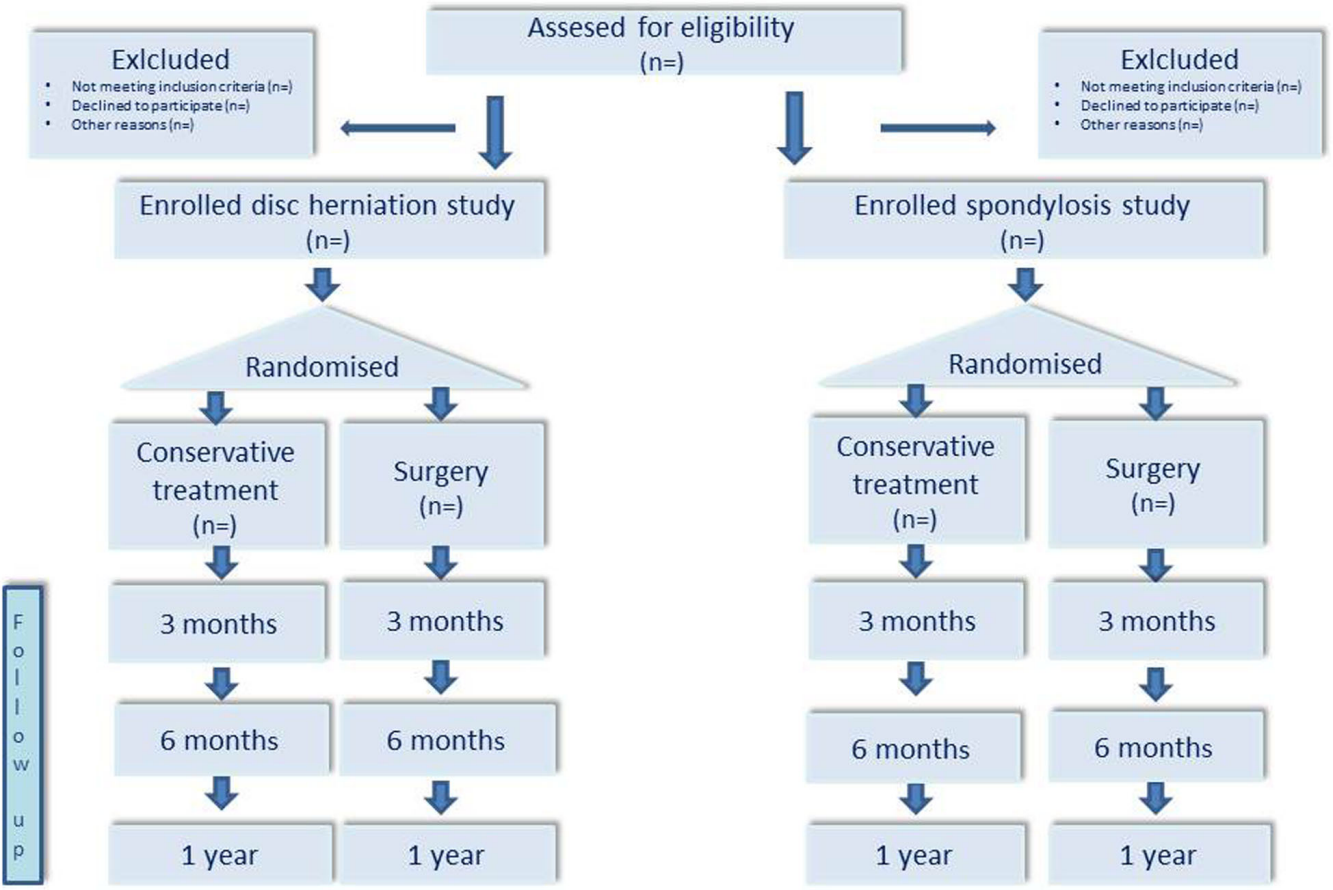
**Fig. 1** Study flow diagram Disc herniation study: one level disc herniation (C5/6 or C6/7) Spondylosis study: one or two level spondylosis (C5/6 and/or C6/7)

The primary aim of this study is to compare the effectiveness of surgical and nonsurgical treatment in patients with cervical radiculopathy through two separate trials, one including disc herniation and the other including spondylosis (Fig. 1). Secondary aims are to evaluate the cost-effectiveness and factors that predict the success of the two treatments, along with exploring the success rate and expectations of patients by asking them to fill in their expected primary outcome score at the baseline.

### Specific aims

1. To test the hypothesis that the effectiveness of surgery measured by the mean difference in Neck Disability Index (NDI) and arm pain – adjusted for the baseline at a 1-year follow-up – in patients with cervical radiculopathy does not differ from nonsurgical treatment in:

- Study 1: one-level disc herniation (C5/6 or C6/7)
- Study 2: one- or two-level spondylosis (C5/6 and/or C6/7)

2. To test the hypothesis that surgery is more effective in patients with more clinical findings (dermatomal sensory loss, myotonal weakness, and reflex disturbance) at the baseline.
3. To estimate the cost-effectiveness for healthcare costs and societal costs (including sickness absence) in surgical versus nonsurgical patients.
4. To assess radiological (MRI and CT) measurements of the foraminal area and nerve compression, and whether morphological changes at the 1-year mark can predict clinical changes (NDI and arm pain).
5. To evaluate treatment-outcome expectations by asking patients to fill in their expected improvements at the baseline, and to compare these with previously published MCIC values and outcomes at the 1-year mark.

## Methods

### Design

This study is designed as two randomised controlled trials comparing cervical decompression and non-operative treatment with cost-effectiveness analysis and the assessment of expectations and predictors of outcomes. The main research question will be evaluated through a one-year follow-up. The trial is registered with the Norwegian ethics committee, REK 2017/2125, and in Clinical Trials – as NCT03674619 – on September 17, 2018. The trial follows the recommendations put forth by SPIRIT (Standard Protocol Items: Recommendations for Interventional Trials) [32].

### Patients

All patients referred to Oslo University Hospital for treatment of cervical radiculopathy – from levels C5/C6 and C6/C7 – will be screened for inclusion in the study. The patients are primarily referred by their general practitioners, private clinics, or neurology departments at other hospitals in the Health Region South-Eastern Norway, covering a population of about 2.9 million inhabitants.

#### Inclusion criteria

This definition of cervical radiculopathy is according to a previously described minimum criteria set [5]. The inclusion criteria are listed in Table 1.

**Table 1:** Inclusion criteria

• Aged 20 to 65 years.	
• Study 1: Neck and arm pain for at least 3 months, and a corresponding herniation involving one cervical nerve root (C6 or C7) Study 2: Neck and arm pain for at least 3 months, with corresponding spondylosis involving C6 and/or C7	
• Arm pain intensity of at least 4 on a scale from 0 (no pain) to 10 (worst possible pain)	
• Willing to accept either of the treatment alternatives	
• Neck Disability Index (NDI) > 30%	

The patients will be informed about the study in detail, both through a standardised written text and orally. They will be informed about what is already known, including the natural course and the effectiveness of the two interventions.

#### Exclusion criteria

Patients with any previous cervical fractures or cervical spine surgery; signs of myelopathy; rapidly progressive paresis or paresis < grade 4; pregnancy; arthritis involving the cervical spine; infection or active cancer; generalised pain syndrome; serious psychiatric or somatic disease that excludes one of the treatment alternatives; concomitant shoulder disorders that may interfere with the outcome; abuse of medication/narcotics; inability to understand written Norwegian; and unwillingness to accept one of the treatment alternatives.

### Randomisation

The patients will be randomized using Viedoc electronic randomisation. This is an independent institution that uses permuted blocks that are unknown to the patient coordinator, which will randomise patients in study A and B. The randomisation process is unknown to the patient coordinator, who is not involved in treatment and evaluation. The coordinator will accordingly make treatment appointments.

### Blinding

The outcome assessor and the statistician will be blinded to treatment allocation. The data will be extracted in an unidentifiable manner and will not contain any information that can reveal what treatment a single subject or a group were randomised to.

### Interventions

All the interventions will commence within 2–3 weeks after randomisation.

#### Decompression surgery

A recent systematic review that included 39 randomised controlled trials concluded that the surgeon, patient, and healthcare provider can choose any surgical technique based on experience, preference, and cost [33]. In the present study, we plan to use anterior discectomy, which is the most commonly used procedure at the neurosurgical department of Oslo University Hospital. This strategy is supported by a recent randomised controlled trial at our hospital that found no clinical benefits of disc prosthesis [22]. All operations will be carried out by experienced and qualified neurosurgeons.

Anterior discectomy will be performed, and a microscope will be used. After separation of the platysma muscle, the pre-vertebral space is reached by an approach medial to the sternocleido-mastoid muscle and carotid artery, and lateral to the trachea and oesophagus. Subsequently, the disc is incised and the corpora are distracted to perform discectomy. Usually, the posterior ligament is cut, the spinal root is decompressed, and – if necessary – the arthritic rims are removed. An inter-vertebral fusion device then is inserted. Two levels are allowed in the spondylosis study.

#### Nonsurgical intervention

Patients will first meet an experienced specialist in physical medicine and rehabilitation, who will answer their concerns and questions, and repeat the information given before inclusion, if necessary. The aim of this brief intervention is to promote a better understanding and coping with the condition. The intervention will further include supervision by a physiotherapist (six sessions altogether), who will provide advice on how to handle secondary neck muscle pain and dysfunction, reduce eventual fear behaviour, and provide advice to stay active.

#### Crossover

Study subjects randomised to nonsurgical intervention will have the option of crossing over to surgery. They will simply have to express their wish to cross over to the treating physician or physiotherapist. The expected reasons for cross overs are a rapid decline in the neurological status or unbearable pain in the arm. The surgeon will then conduct a new assessment and, after a short observation period, these patients will be offered the option of surgery.

### Outcome measurements

Baseline data will be obtained before randomisation and 12, 26, and 52 weeks after randomisation.

*The primary outcomes are*:

- The Neck Disability Index, which consists of ten questions about pain-related disability, including items such as headaches, concentration problems, reading issues and sleep disturbances. Each item is rated by choosing one of five response categories, and then transformed into a total score ranging from 0 to 100 (worst possible). The Norwegian version has been validated in patients with neck pain and with cervical radiculopathy [34, 35].
- Arm pain, measured by a Numeric Rating Scale (NRS) from 0 (no pain) to 10 (worst imaginable pain) [36].
- Follow-up at 52 weeks is the primary endpoint.

*The secondary outcomes are:*

- Neck pain, measured by a Numeric Rating Scale (NRS) from 0 (no pain) to 10 (worst imaginable pain).
- Perceived recovery or change of the main symptoms, rated on a numeric scale ranging from − 9 (worst possible change) to 9 (best possible change) [37].
- EuroQol (EQ-5D and EQ-VAS). EQ-5D includes five facets: mobility, self-care, daily activities, pain/discomfort, and anxiety/depression, and each has three response categories. The responses are transformed into and indexed to value the patients’ health-related quality of life for a cost–utility analysis. Patients score their health from 0 (as bad as possible) to 100 (best possible) by EQ-VAS. The Norwegian version has been validated in patients with back pain, idiopathic scoliosis, and cervical radiculopathy [35, 38, 39].
- Fear-avoidance beliefs, evaluated using the Fear Avoidance Beliefs Questionnaire (FABQ) [40–43].
- Emotional distress will be assessed by the 10-question version of the Hopkins Symptom Check List (HSCL-10) [44, 45].
- Medicine consumption the week before inclusion and the week before each follow-up will be registered.
- Sickness absence data will be collected from the National Social Security Institution for the year before and after inclusion.
- Dysphagia [46].
- Frequency of complications (dural tears, and disturbances of the larynx recurrent nerve, index-level nerve, oesophagus, trachea, or large vessel).
- Frequency of reoperation after surgery and frequency of operations in patients allocated to nonsurgical treatment.
- Other treatments. We will register concomitant care and interventions. There are no restrictions related to concomitant care.
- Exploring global success rates by asking the patients about how their arm and neck pain are compared to prior to treatment (ranging from much worse to much better).
- Patient expectations. Exploring patient expectations ahead of treatment. The patients are asked to fill out the Neck Disability Index – as if they were at 52 weeks post-treatment – and selecting the lowest category they would be content with for each item. The patients are also asked to report what they expect their symptoms to be like in 52 weeks (ranging from much worse to much better), registered for arm pain, neck pain, and headaches separately.

Outcomes of the predictor and cost–utility analyses are briefly outlined in Tables 2 and 3, respectively.

**Table 2:** Predictors study

Neurological (sensory abnormality and weakness) [5]	
MRI findings (disc herniation, spondylosis, and number of levels involved)	
Sickness absence [9]	
Patient expectations [47]	
Emotional distress	
Fear-avoidance beliefs	
Age	
Gender	
Smoking	
Severity of primary outcome at the baseline	
*Primary outcome*	
Change in the primary outcome of the trial	
Number of patients recovered (mean pain scores 0, 1, and 2) or reached the expected outcome at 52 weeks	
Number of patients working full time at the 1-year mark (adjusted for absence at the baseline)

**Table 3:** Cost–utility study

*Direct costs*^*a*^,	
• Surgery	
- Direct surgical costs	
- Implants	
- Hospital stay, including eventual complications and emergencies	
• Nonsurgical treatment	
- Consultation	
- Physiotherapy	
• Both groups	
- Medication	
- Consultations	
- Imaging	
*Indirect costs*	
Sickness absence [9]	
*Utility*	
EQ-5D	

^a^A sensitivity analysis of costs will estimate direct costs using prices at private clinics in Norway

Outcomes of the radiology study and the expected-outcome study are briefly outlined in Table 4.

**Table 4:** Radiology and expected-outcome study

*Radiology*	
CT at the baseline and at 1-year follow-up for assessment of foraminal area	
MRI at the baseline and 1-year follow-up for assessment of nerve-root compression and dislocation	
*Expected outcome*	
Expected NDI, NRS for neck and arm pain for willingness to undergo surgery.	
Expected NDI, NRS for neck and arm pain for willingness to undergo nonsurgical treatment.	

The timing of the outcome measurements is outlined in Table 5.

**Table 5:** Outcome measurements

Outcome	When they will be evaluated			
	Baseline	12 weeks	26 weeks	52 weeks
Primary:				
NDI	x	x	x	x
Arm pain (NRS)	x	x	x	x
Secondary:				
Neck pain (NRS)	x	x	x	x
Patient expectations	x			
Perceived recovery		x	x	x
Success rate		x	x	x
EuroQol (EQ-5D and EQ-VAS)	x	x	x	x
FABQ	x	x	x	x
HSCL-10	x	x	x	x
Medicine consumption	x	x	x	x
Sickness absence	x	x	x	x
Dysphagia	x	x	x	x
Frequency of surgical complications		x	x	x
Frequency of reoperations		x	x	x
Cross overs in the nonsurgical group		x	x	x
Patient demographics	x			
Neurological status, incl. Grip strength	x	x	x	x

### Sample size

Including 36 patients in each treatment group is estimated to have 80% power for detecting a clinically significant difference (*p* < 0.05) in NDI at a 1-year follow-up of 12, assuming a standard deviation of 18. Assuming a 10% drop-out and 20% cross-over rate, we plan to include 50 patients in each group in each trial, giving a total of 200 patients for evaluation of predictors.

### Data analysis

The principle of ‘intention to treat’ will be applied for the primary analyses comparing outcomes between groups. We are also planning to perform an analysis based on the ‘as treated’ principle. Multiple imputations will be used for the primary outcome if missing data exceeds 10%. ANCOVA or multiple-regression analysis will be used to compare outcomes among the different groups at the 1-year mark, adjusting for respective outcome variables at the baseline. In addition, we will use mixed models to investigate changes over time. Categorical variables will be assessed with Pearson’s chi-square test or logistic regression. Results will be presented as mean differences or as odds ratios for categorical data, both with 95% confidence intervals (95% CI). Sensitivity analyses, including those with treatments performed according to the protocol, will be added. A non-parametric bootstrapping technique will be used for health economic analyses. Kappa and limits of agreement will be estimated, and logistic and linear regression will be applied to analyse radiology. Predictors will be analysed using multiple-linear and logistic regression. The expected primary outcomes will be calculated for each patient; mean values (95% CI) will be estimated.

### Implementation and study group

The project will involve all the departments (neurosurgery, neurology, and physical medicine and rehabilitation) treating patients with cervical radiculopathy at Oslo University Hospital (OUS). Because the population covered by OUS is about 2.9 million, we have decided to conduct the study at OUS and not involve other neurosurgical institutions in Norway. All referrals will be coordinated from the respective units, and possible candidates for inclusion will be evaluated by a specialist in physical medicine and rehabilitation (doctoral candidate), along with an experienced neurosurgeon (doctoral candidate or equivalent). Only patients with cervical radiculopathy will be included in the study. Neck pain itself, even accompanied by radiological findings, is not an indication for surgery. A research nurse will handle patients found to be eligible after oral and written consent is obtained. The study group consists of experienced researchers who have expertise in conducting large, high-quality, randomised controlled trials. Surgeries will be performed by an experienced neurosurgeon and non-operative treatment by an experienced specialist in physical medicine and rehabilitation. The head of physiotherapy, along with physiotherapists who are currently conducting non-operative treatments of these patients at the physical medicine and rehabilitation department, have been engaged in the planning and running of the non-operative treatment. Two study secretaries at the neurosurgical department have coordinated another RCT at the department with great success, which is of importance for the quality of the current project.

Patients included in the planned trial are all surgical candidates with high expectations for surgery. The physicians recruiting the patients must be trained to inform the patients about the lack of evidence of surgery, and that the trial is being conducted to ascertain the best choice in the future. This is important when recruiting patients, to reduce bias in favour of surgery.

### Client participation

When designing this trial, we discussed several issues with the former leader of the Norwegian Patient Organisation for Spinal Pain. They are fully informed about the project. They will be involved in further interpretation and implementation of the study, preferably in collaboration with other patients who have undergone neck surgery.

### Data collection

The designated investigator staff will enter the data required by the protocol into eCase report forms (eCRF). The investigator is responsible for assuring that data entered into the eCRF is complete and accurate, and that entries are made in a timely manner. The Clinical Data Management System (CDMS) used for eCRF in this study is ViedocTM. The setup of the study-specific eCRF in the CDMS will be performed by the Clinical Trial Unit, Research Support Services, Oslo University Hospital. After database locking, the investigator will receive a digital copy of the subject data for archiving at the investigation site.

### Database management

Data management will be performed by the Clinical Trial Unit, Research Support Services, Oslo University Hospital. The data-management procedures will be performed in accordance with the department’s Standard Operating Procedures (SOPs) and International Council for Harmonisation (ICH) guidelines. The data-management process will be described in the study-specific data handling plan and the study-specific data handling report after database closure.

After database closure, the data will be stored in a dedicated and secured area at OUS. Data will be stored with all identifiers removed, where each study participant can only be recognised by his/her unique trial subject number.

### Ethics and dissemination

We have been granted approval by The Committee of Medical Ethics in Health Region South-Eastern Norway and from The Research Board at Oslo University Hospital, Norway. Standard informed written consent will be obtained from each participant. The trial is formally registered at the National Register, and Clinical Trials and will be reported according to the CONSORT (Consolidated Standards of Reporting Trials) statement [48]. The findings will be published in international journals and presented at national and international conferences. The version number of the current protocol is V.2.0 (07.03.2019). The project is sponsored by the Southern and Eastern Norway Regional Health Authority. The sponsor had no role in designing this trial. A steering committee is responsible for the design and any subsequent amendments to the study protocol. A Data Monitoring Committee (DMC), independent from the sponsors of the study, has full insight into preliminary data. The DMC has the power to terminate the trial in case the interim results suggest a need for this.

## Discussion

Published protocols include a Dutch trial comparing the effectiveness of surgical treatment and a wait-and-see approach including information about the nature and prognosis of the problem along with trustworthy counselling in patients with cervical radiculopathy caused by disc herniation [16]. In addition, a Swedish trial compares the effectiveness of a comprehensive neck-specific training regimen combined with an additional cognitive behavioural approach to prescribed physical activity [49]. The results from the trials, including the current protocol, are likely to improve our understanding of the treatment and prognosis of patients with cervical radiculopathy. The prognostic part of the present study will preferably be able to detect certain patient characteristics that may aid in improving indications. Finally, the cost–utility analysis is important to estimate the healthcare and societal costs, which may help to better distribute healthcare resources between surgical and nonsurgical treatment.

## Data Availability

Not applicable.

## Abbreviations

MRI: Magnetic resonance imaging
IL: Interleukin
RCT: Randomised controlled trial
SR: Systematic review
MCIC: Minimal clinical important change
CT: Computed tomography
NDI: Neck Disability Index
REK: Norwegian ethics committee
SPIRIT: Standard Protocol Items Recommendations for Interventional Trials
NRS: Numeric Rating Scale
EuroQol (EQ-5D and EQ-VAS): Health-related quality of life instrument
FABQ: Fear Avoidance Beliefs Questionnaire
HSCL-10: Hopkins Symptom Check List
ANCOVA: Analysis of covariance
OUS: Oslo University Hospital
eCRF: eCase report form
CDMS: Clinical Data Management System
SOPs: Standard Operating Procedures
ICH: International Council for Harmonisation
CONSORT: Consolidated Standards of Reporting Trials
DMC: Data Monitoring Committee
